# Artemisinin derivative artesunate induces radiosensitivity in cervical cancer cells *in vitro* and *in vivo*

**DOI:** 10.1186/1748-717X-9-84

**Published:** 2014-03-25

**Authors:** Judong Luo, Wei Zhu, Yiting Tang, Han Cao, Yuanyuan Zhou, Rong Ji, Xifa Zhou, Zhongkai Lu, Hongying Yang, Shuyu Zhang, Jianping Cao

**Affiliations:** 1 School of Radiation Medicine and Protection and Jiangsu Provincial Key Laboratory of Radiation Medicine and Protection, Soochow University, Suzhou 215123, China; 2 Deaprtment of Radiotherapy, Changzhou Tumor Hospital, Soochow University, Changzhou 213001, China; 3 School of Radiation Medicine and Protection, Medical College of Soochow University, 199 Ren’ai Rd, Suzhou 215123, China

**Keywords:** Artesunate (ART), Cervical cancer, Radiosensitivity, Cell cycle, Apoptosis

## Abstract

**Objective:**

Cervical cancer is the third most common type of cancer in women worldwide and radiotherapy remains its predominant therapeutic treatment. Artesunate (ART), a derivative of artemisinin, has shown radiosensitization effect in previous studies. However, such effects of ART have not yet been revealed for cervical cancer cells.

**Methods:**

The effect of ART on radiosensitivity of human cervical cancer cell lines HeLa and SiHa was assessed using the clonogenic assay. Cell cycle progression and apoptosis alterations were analyzed by flow cytometry. For *in vivo* study, HeLa or SiHa cells were inoculated into nude mice to establish tumors. Tissues from xenografts were obtained to detect the changes of microvessel density, apoptosis and cell cycle distribution. Microarray was used to analyze differentially expressed genes.

**Results:**

ART increased the radiosensitivity of HeLa cells (SER = 1.43, *P* < 0.001) but not of SiHa cells. Apoptosis and the G2-M phase transition induced by X-ray irradiation (IR) were enhanced by ART via increased Cyclin B1 expression in HeLa cells. Tumor growth of xenografts from HeLa but not SiHa cells was significantly inhibited by irradiation combined with ART (tumor volume reduction of 72.34% in IR + ART group *vs.* 41.22% in IR group in HeLa cells and 48.79% in IR + ART group *vs.* 44.03% in IR alone group in SiHa cells). Compared with the irradiated group, cell apoptosis was increased and the G2/M cell cycle arrest was enhanced in the group receiving irradiation combined with ART. Furthermore, compared with radiation alone, X-ray irradiation plus ART affected the expression of 203 genes that function in multiple pathways including RNA transport, the spliceosome, RNA degradation and p53 signaling.

**Conclusion:**

ART potently abrogates the G2 checkpoint control in HeLa cells. ART can induce radiosensitivity of HeLa cells *in vitro* and *in vivo*.

## Introduction

Cervical cancer used to be the number 1 killer of female human beings who suffered from cancer, with the development of general survey technique, the morbidity of cervical cancer decreased recently. However, cervical cancer remains the third most common type of cancer in women worldwide, especially in the medical underdeveloped areas, and radiotherapy is a common treatment for this gynecological malignancy [1]. Radiotherapy for cancer of the cervix can be external or/and internal, which treats cervical cancer by using high-energy rays and destroy the cancer cells while doing as little harm as possible to normal cells. However, radioresistance remains one of the major reasons for clinical failure of radiotherapy [2]. Thus, to improve the efficacy of radiotherapy for cervical cancer, the combination of radiation with additional radiosensitizing agents is needed.

Artemisinin, a chemical compound derived from the sweet wormwood plant (Artemisia annua), has been well established to successfully treat malaria and viruses in humans for years. Various derivatives of artemisinin, including artesunate, artemether, dihydroartemisinin (DHA) and arteether have been identified [3,4]. Artesunate (ART) is a semi-synthetic derivative of artemisinin and a more effective antimalarial agent [5]. ART reveals remarkable activity against otherwise multidrug-resistant Plasmodium falciparum and P. vivax malaria. ART has also showed anticancer activity against a variety of cancer cells, especially in leukemia and colon cancer cell lines. The anticancer activity of ART is associated with multiple mechanisms, including reactive oxygen species (ROS), oxidative DNA damage, sustained DNA double-strand breaks and apoptosis [6-8].

Artemisinin and its derivative dihydroartemisinin have shown radiosensitizing effect in cervical cancer cells [9,10]. Recently, the ART has been implicated as an effective radiosensitiser in glioblastoma cells by decreasing survivin expression [11] and in lung cancer cells via increasing NO production [12]. However, whether such an effect of ART exists in cervical cancer cells remains elusive. This study aims to investigate the radiosensitizing effects of ART and underlying mechanisms *in vitro* and *in vivo*. Our results show that artesunate selective radiosensitization of human cervical cancer HeLa cells through abrogation of radiation-induced G2 block and cell apoptosis.

## Materials and methods

### Reagents and cell culture

ART was purchased from Sigma Chemical Co. (Sigma Chemical Co: St. Louis, MO) and was dissolved in dimethylsulfoxide (DMSO, Solon, OH) to 10 mmol/L as stock solution (−20°C stocked), and diluted by DMEM to final concentration. For *in vivo* studies, ART was diluted with sterile PBS at 5 mg/ml before each administration.

The human cervical cancer cell lines HeLa and SiHa were kind gifts from Prof. Saijun Fan, Georgetown University. These cells were maintained in DMEM supplemented with 10% FBS and antibiotics (100 units/ml penicillin G, 100 units/ml streptomycin sulfate; Gibco, Grand Island, NY). Cells were grown in a 37°C incubator with 5% CO_2_.

### Cytotoxicity assay

Cells (2 × 10^3^) were seeded into 96-well plates in 100 μl of DMEM medium and were incubated for 24 h, and then the cells were treated with indicated concentrations of ART followed by incubated with 200 μg/ml MTT (3-(4,5-dimethylthiazol-2-yl)-2,5-diphenyl-2H-tetrazolium bromide, Sigma) for 4 h. The reaction product was dissolved in DMSO. Absorbance was measured at background wavelength of 570 nm, reference wavelength of 630 nm using a microplate reader. Three independent experiments were done in triplicate.

### Clonogenic assay

Clonogenic assay was performed as described previously [10]. Cells were seeded into six-well plates at 500–2,000 cells/well depending on the dose of radiation. Twenty-four hours after seeding, cells were treated with ART or DMSO for 24 h. Cells were exposed to various doses (0, 2, 4, 6 and 8 Gy) of X-rays irradiation from linear accelerators (Varian, USA) at a dose rate of 2 Gy/min; a 1.5-cm bolus was used as a compensator. After radiation, drug-containing media was immediately replaced by fresh DMEM. The cells were then grown from 7–12 days to allow for colony formation and subsequently fixed and stained using crystal violet. Colonies consisting of 50 or more cells were counted as clones.

### Measurement of apoptosis

Cells were treated with ART for 24 h prior to treatment with 2 or 6 Gy irradiation. Apoptosis was measured using propidium iodide (PI)/Annexin-V double staining following manufacturer’s instructions (Keygen Biotech, Nanjing, China). Cells were harvested 24 h after treatment with ART; apoptotic fractions were measured using flow cytometry (Beckman, USA). The Annexin-V+/PI- cells are early in the apoptotic process, the Annexin-V+/PI + cells indicating late apoptosis. The percentage of both kinds of cells was counted. The Annexin-V-/PI + cells are considered to be necrotic cells.

For tissue samples, 5 μm xenograft sections were deparaffinized in xylene and hydrated in decreasing concentrations of ethanol, and the terminal deoxynucleotidyl transferase dUTP nick-end labeling (TUNEL) assay was performed following manufacturer’s instructions (Keygen Biotech, Nanjing, China). Ten random fields from 4 slides per group were examined. TUNEL-positive brown nuclei within tissues were counted. Data were expressed as the percentage of apoptotic cells per field.

### Cell cycle progression analysis

Cells were treated with ART for 24 h. Cells were then changed with fresh medium and irradiated at the indicated doses. 24 h after irradiation, both floating and attached cells were harvested and analyzed using the procedures described previously (10). For flow cytometry, 10,000 cells per sample were collected (Beckman, USA).

For cell cycle analyses of tissue samples, tissue specimens were taken from nude mice and mixed with 200 μl 0.25% trypsin and EDTA (1:1), stirred 1 min at room temperature and then filtered with a 70 μm nylon net. Tumor cells were collected and pooled with the cells floating in the medium. Cell suspensions were centrifuged 5 min at 1,500 rpm, room temperature, then washed and fixed with ethanol at 4°C overnight. All samples were then washed with PBS and resuspended in PI (50 μg/mL) and RNase A (20 μg/mL) in PBS for 30 min at room temperature. Stained cells were analyzed by flow cytometry (Beckman, USA).

### Western blot

Cells were treated for 24 h with ART. Cells were then changed with fresh medium and irradiated at the indicated doses. The cells were then washed twice with ice-cold PBS and directly lysed in 200 μl of cell lysis buffer. Tumors from nude mice were resected, homogenized and lysed. Western blot was performed as described previously [13]. Primary antibodies against Wee1, Cyclin B1, P53 and Cdc2 were all obtained from Santa Cruz Biotechnology (Santa Cruz, CA) and used at a 1:1,000 -1:2,000 dilution. β-Actin was used as the loading control and detected using a mouse monoclonal anti-β-actin antibody (Santa Cruz Biotechnology).

### Xenograft studies of nude mice

Four-week-old male outbred BALB/c mice were purchased from Shanghai SLAC Laboratory Animal Co., Ltd. (Shanghai, China), and kept under specific pathogen-free conditions. HeLa or SiHa cells (2 × 10^6^) were suspended in 100 μl PBS and then inoculated subcutaneously into each posterior flank region of BALB/c nude mice. When the average volume of tumor achieved 5 mm × 5 mm × 5 mm, mice were randomized into four groups as follows (5 animals per group): 1) Control group (no radiation, injection of 200 μl sterile PBS once a day for 7 days), 2) ART alone group (no radiation, injection of ART once a day for 7 days at 100 mg/kg/day with a total volume of 200 μl), 3) Radiation (IR) alone group (injection of 200 μl sterile PBS for 7 days and radiated at a dose of 10 Gy at the 7th day one hour after the last PBS was injected, or 4) IR plus ART treatment group (injection of 200 μl ART once a day for 7 days at 100 mg/kg/day and radiated at a dose of 10 Gy on the 7 th day after ART treatment). Both PBS and ART were intraperitoneal injected.

Tumor diameters were measured at regular intervals with digital calipers, and the tumor volume in mm^3^ was calculated using the formula: volume = (width)^2^ × length/2. A tumor growth curve was constructed and data are presented as means ± SEM. Animals were sacrificed 21 days after the first inoculation. All the animal study were approved by the Animal Experimentation Ethics Committee of Soochow University.

### Immunohistochemistry (IHC)

Immunohistochemistry of CD 31 was performed as described previously [13]. Fetal lung tissue was used as a positive control, and omission of primary antibody was performed as a negative control.

### Microarray analysis of gene expression

Total RNA was extracted from cells or tissues with Trizol reagent (Invitrogen, Carlsbad, CA). Microarray-based mRNA expression profiling was performed using the Roche-NimbleGen (135 K array) Array (Roche, WI). The microarrays contained approximately 45,033 assay probes corresponding to all of the annotated human mRNA sequences (NCBI HG18, Build 36). Total RNA labeling and hybridization were performed using standard conditions according to manufacturer instructions. Genes with an absolute fold change of 5 or greater were subsequently subjected to pathway analysis using Ingenuity Pathway Analysis (Redwood City, CA).

### Statistical analysis

Data were expressed as the mean ± standard error of the mean (SEM) of at least three independent experiments. Standard error bars are included for all data points. The data were then analyzed using Student’s t-test when only two groups were present, or assessed by one-way analysis of variance (ANOVA) when more than two groups were compared. The interaction between ART and radiation was tested using two-way ANOVA. The sensitizer enhancement ratios (SER) were measured using Sigmaplot software according to the multi-target single hit model. Differences were considered statistically significant when *P* < 0.05.

## Results

### ART induces cytotoxicity in human cervical cancer cells

**Figure 1:**
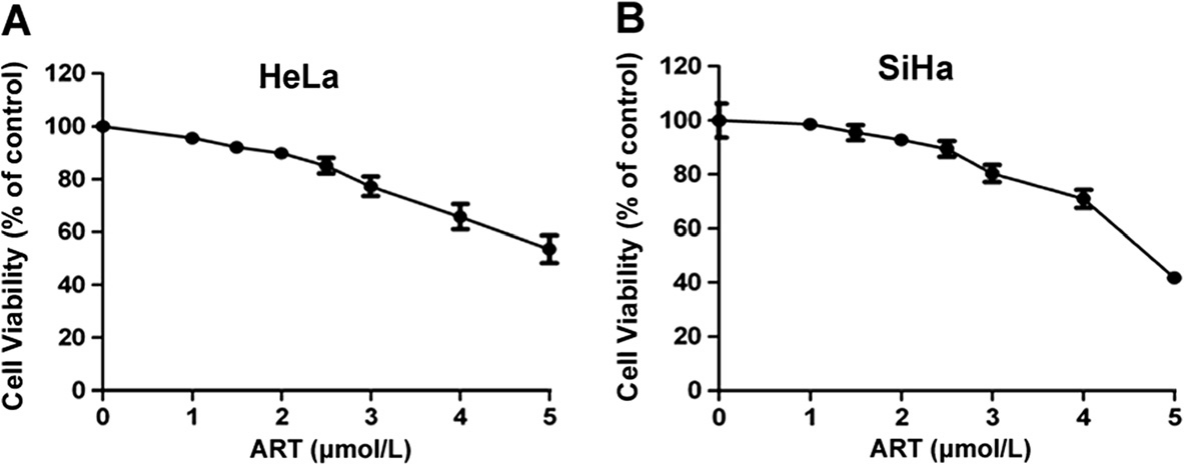
**Cytotoxicity of ART on HeLa and SiHa cells.** The ART-induced cytotoxicity in **(A)** HeLa and **(B)** SiHa cells. HeLa and SiHa cells were exposed to indicated concentrations of ART for 24 h. Cell survival was assessed using an MTT assay. The data are shown as the mean values ± the standard error of the mean (SEM) for three independent experiments.

To evaluate the anticancer effect of ART on cultured human cervical cancer cells, HeLa and SiHa cells were treated with different concentrations of ART. The OD value of cells treated without ART were regarded as control (100%) and then the cell groups treated with different concentration of ART were versus with control. The MTT assay revealed that the inhibitory effects elicited by ART were dependent on its concentration (Figure 1A and B). The fifty percent inhibition concentration (IC50) of ART against HeLa and SiHa cells was 5.47 μmol/L and 6.34 μmol/L, respectively. To evaluate the sensitizing ability of ART on cervical cancer cells to irradiation, moderately toxic doses that reduced cell viability to approximately 90% were used. ART induced approximately 10% inhibition of HeLa cell viability with the concentration of 2.0 μmol/L, which was comparable to the effect of 2.5 μmol/L ART on SiHa cells. These concentrations were chosen for subsequent experiments.

### ART increases the radiosensitivity in HeLa but not SiHa cells

**Figure 2:**
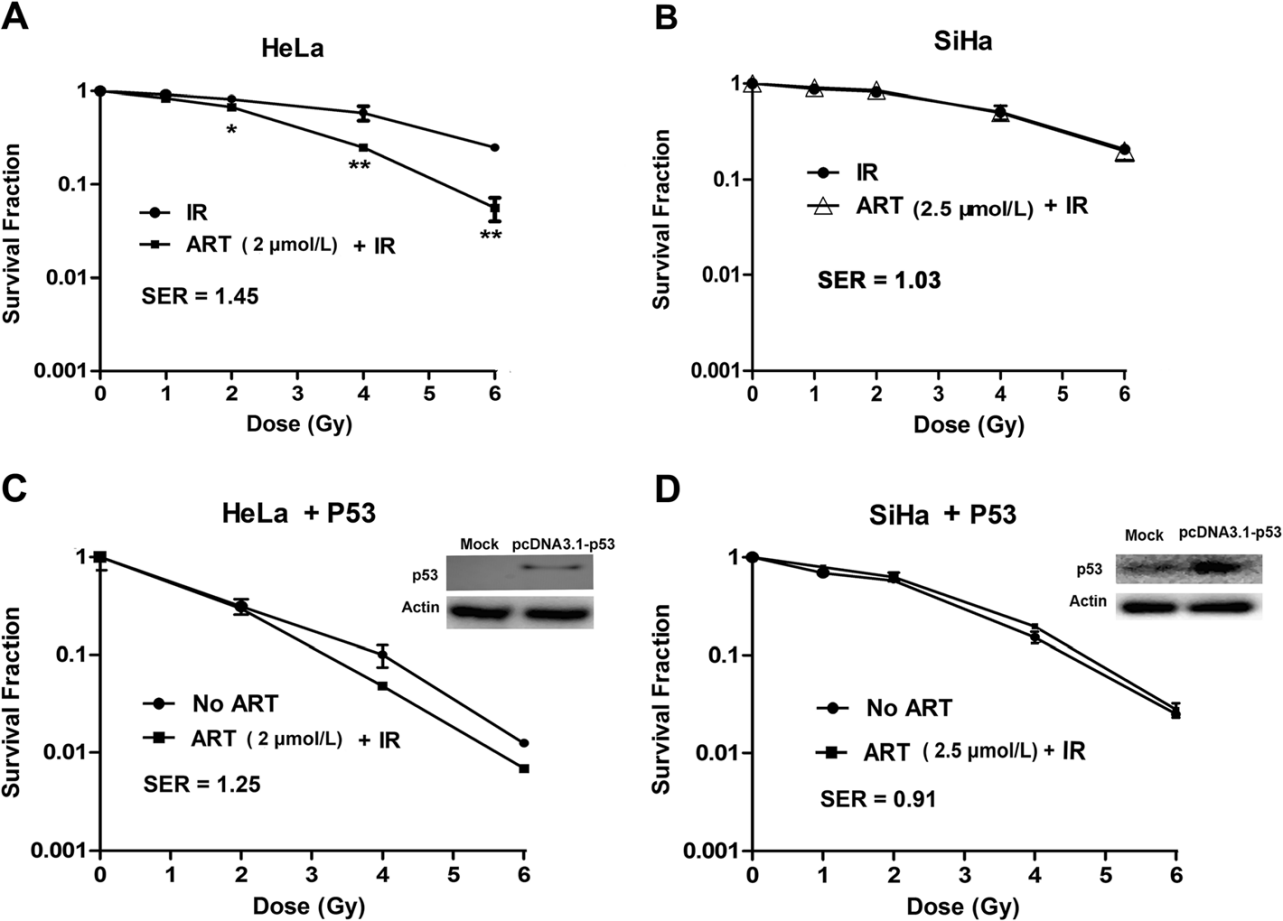
**The ART-induced radiosensitivity in HeLa (A) and SiHa (B) cells. (C)** and **(D)** The ART-induced radiosensitivity in wild-type p53-overexpression HeLa and SiHa cells. Western blot of p53 and internal control in pcDNA3.1- or pcDNA3.1-p53-transfected HeLa **(C)** and SiHa **(D)** cells. Clonogenic cell survival curves were generated for HeLa and SiHa cells that were treated with the indicated concentrations of ART for 24 h and then were exposed to 2, 4, 6 or 8 Gy IR. The survival data were normalized to those of the unirradiated control group. The data are shown as mean ± SEM for three independent experiments. The SER was calculated for HeLa or SiHa cells that were treated with 2.0 or 2.5 μmol/L ART prior to X-ray irradiation according to the multi-target single hit model. Values shown are the mean ± SEM for three independent experiments. *P < 0.05; **P < 0.01, compared with irradiated cells.

To investigate the influence of ART on the radiosensitivity of HeLa and SiHa cells, we performed an *in vitro* clonogenic cell survival assay. HeLa cells pretreated with 2.0 μmol/L ART plus X-ray irradiation exhibited significantly lower clonogenic survival fractions than cells treated with radiation alone. The sensitizer enhancement ratio (SER) was 1.43 for cells treated with radiation plus ART, compared to cells treated with radiation alone (Figure 2A). SiHa cells treated with radiation plus 2.5 μmol/L ART exhibited a SER of 1.03 (Figure 2B). The data were further analyzed using two-way ANOVA to test the interaction effect between ART and radiation. Our results indicated that interaction between ART and radiation was statistically significant (*P* < 0.001) for HeLa cells, suggesting that ART treatment sensitized cells to X-ray irradiation. However, in SiHa cells, the interaction effect between ART and radiation was not statistically significant (*P* > 0.05). Taken together, these results demonstrated that treatment with ART could increase the radiosensitivity of human HeLa cells but not of SiHa cells.

We further investigated whether the difference between HeLa and SiHa cells in the ART-induced radiosensitizing effect is due to p53 status and expression level. HeLa cells with mutant p53 were transfected with a p53-overexpression vector (pcDNA3.1-p53). As shown in Figures 2C, introduction of wild-type p53 could partially reduce the radiosensitivity caused by ART (SER = 1.25) in HeLa cells, indicating that p53 status is likely to be one of the reasons for the selective radiosensitizing effect of ART. However, forced overexpression of p53 in SiHa cells significantly reduced the radiosensitizing effect of ART (SER = 0.91, Figure 2D), indicating different roles of p53 in the two types of cell lines.

### ART induces apoptosis and necrosis in Hela cells

**Figure 3:**
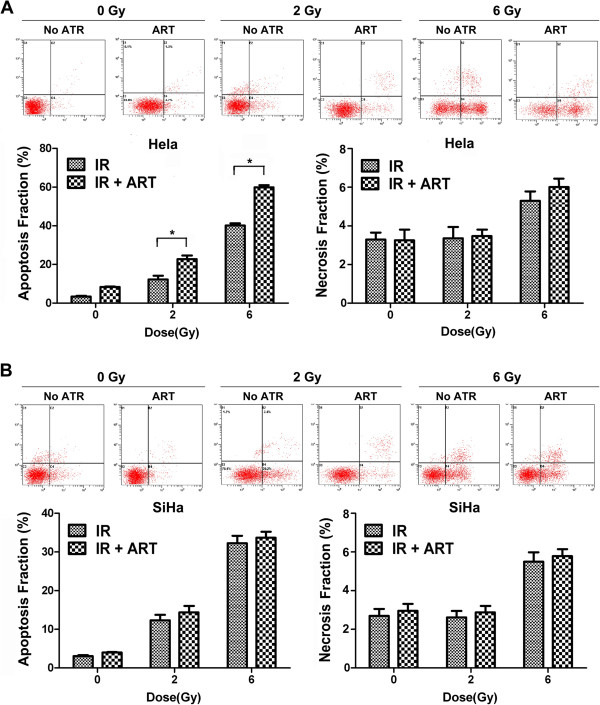
**Induction of apoptosis by ART and radiation in HeLa and SiHa cells.** Cells were treated with ART for 24 h prior to treatment with 2 or 6 Gy irradiation. Apoptosis and necrosis were measured using propidium iodide (PI)/annexin-V double staining in **(A)** Hela and **(B)** SiHa cells. The data are shown as mean ± SEM for three independent experiments. Statistical analysis between the groups was determined by ANOVA; *P < 0.05.

We next investigated whether reduced clonogenic survival upon combined treatment with ART and X-ray irradiation was associated with increased apoptosis and necrosis. As shown in Figure 3A and B, 6Gy X-ray irradiation induced apoptosis (Annexin-V+/PI- plus Annexin-V+/PI + cells) and necrosis (Annexin-V-/PI + cells) in both HeLa and SiHa cell lines, and 2Gy X-ray irradiation only induced apoptosis but not necrosis in the two cell lines. This is mainly because necrosis arises when exposed to high dosage. ART treatment further enhanced the apoptosis response of HeLa cells to 2 or 6 Gy of irradiation (IR + ART 22.71% *vs.* IR 12.26% at 2 Gy, *P* < 0.05; IR + ART 59.92% *vs.* IR 40.08% at 6 Gy, *P <* 0.05, Figure 3A). However, there is no difference of the apoptosis response between radiation plus ART and radiation alone group in SiHa cells (IR + ART 14.35% *vs.* IR 12.276% at 2 Gy, *P* > 0.05; IR + ART 33.67% *vs.* IR 32.25% at 6 Gy, *P* > 0.05). The percentage of necrotic cells was similar between ART-treated and control cells in both cell lines (Figure 3A and B). Taken together, these results demonstrate that ART augments apoptotic cell death of HeLa cells in response to irradiation.

### ART combined with X-ray irradiation modulates cell cycle progression

**Figure 4:**
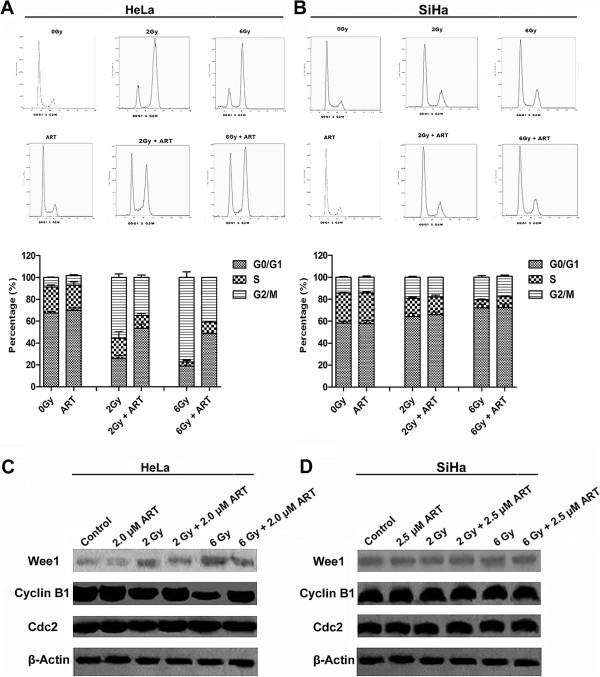
**The effect of ART and radiation on HeLa (A) and SiHa (B) cell cycle progression.** Cells were treated with or without 2.0 μmol/L (HeLa) or 2.5 μmol/L ART (SiHa) for 24 h prior to exposure to 6 Gy irradiation (IR). After 24 h, both attached and floating cells were harvested for cell cycle analysis. The expression of Wee1, Cyclin B1 and Cdc2 in HeLa **(C)** and SiHa **(D)** cells. HeLa and SiHa cells were treated with or without ART for 24 h prior to exposure to 6 Gy of X-ray irradiation (IR). The expression levels of Wee1, Cyclin B1, Cdc2 and the internal control β-Actin were evaluated by Western blot.

DNA damage by radiation activates checkpoint pathways that inhibit the progression of cells through G1 and G2 phases and delay progression through S phase. These checkpoints provide cells with enough time to repair damaged DNA prior to resuming cell cycle progression [14,15]. To determine whether the observed ART-induced radiosensitization was associated with changes in cell cycle progression, HeLa and SiHa cells were cultured in DMEM without serum for 24 h prior to the addition of ART, alone or combined with 2 or 6 Gy X-ray irradiation. As shown in Figures 4A and B, radiation induced a G2 arrest in the p53-mutant HeLa cells and a G1 arrest in p53 wild-type SiHa cells. Compared with irradiated cells, cells treatment with ART plus X-ray irradiation showed decreased the population of G2/M arrest in HeLa (but not SiHa) cells (a reduction of 20.77% at 2 Gy and 36.24% at 6 Gy, *P* < 0.05). This result clearly indicates that ART abrogates the G2 checkpoint but elicits no effect on the G1 checkpoint.

As Wee1, Cdc2 and Cyclin B1 play critical roles in the control of the G2/M transition of the cell cycle progression [16-18]. We then investigated whether ART pretreatment modulated the expression of Wee1, Cyclin B1 or Cdc2 after X-ray irradiation of HeLa and SiHa cells. As shown in Figure 4C, 6 Gy X-ray irradiation induced the expression of Wee1. The addition of ART prior to irradiation resulted in decreased Wee1 and increased Cyclin B1 expression in HeLa cells (Figure 4C, left panel). In contrast, relative expression of Cdc2, Wee1 and Cyclin B1 remained unaltered in SiHa cells. These results indicated that combined treatment of ART and radiation reduced Wee1 and increased Cyclin B1 expression, abrogating the G2/M arrest induced by radiation in HeLa cells.

### Irradiation combined with ART inhibits the growth of HeLa xenografts

**Figure 5:**
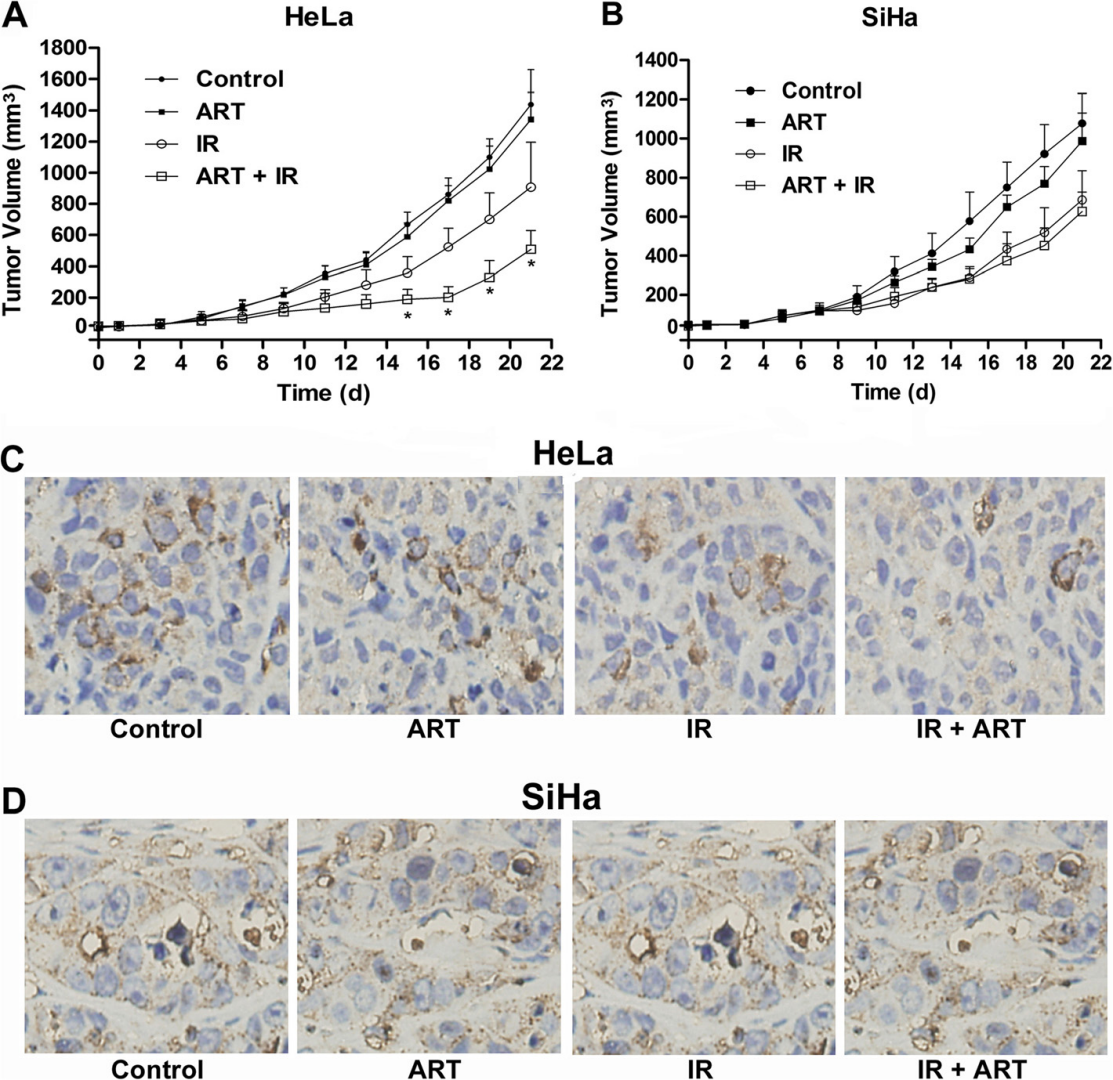
**The effect of ART on the growth and CD31 expression of xenografts.** Each group of mice was composed of five male nude mice. HeLa **(A)** or SiHa cells **(B)** were inoculated under the skin of nude mice. Tumor size was measured at 2 day intervals. *P < 0.05, compared with IR alone group. Data are presented as means ± SEM. Representative IHC showing CD31-stained microvessels in the xenografts from HeLa **(C)** and SiHa cells **(D)**. Scale bar represents 50 μm (magnification, × 400).

**Figure 6:**
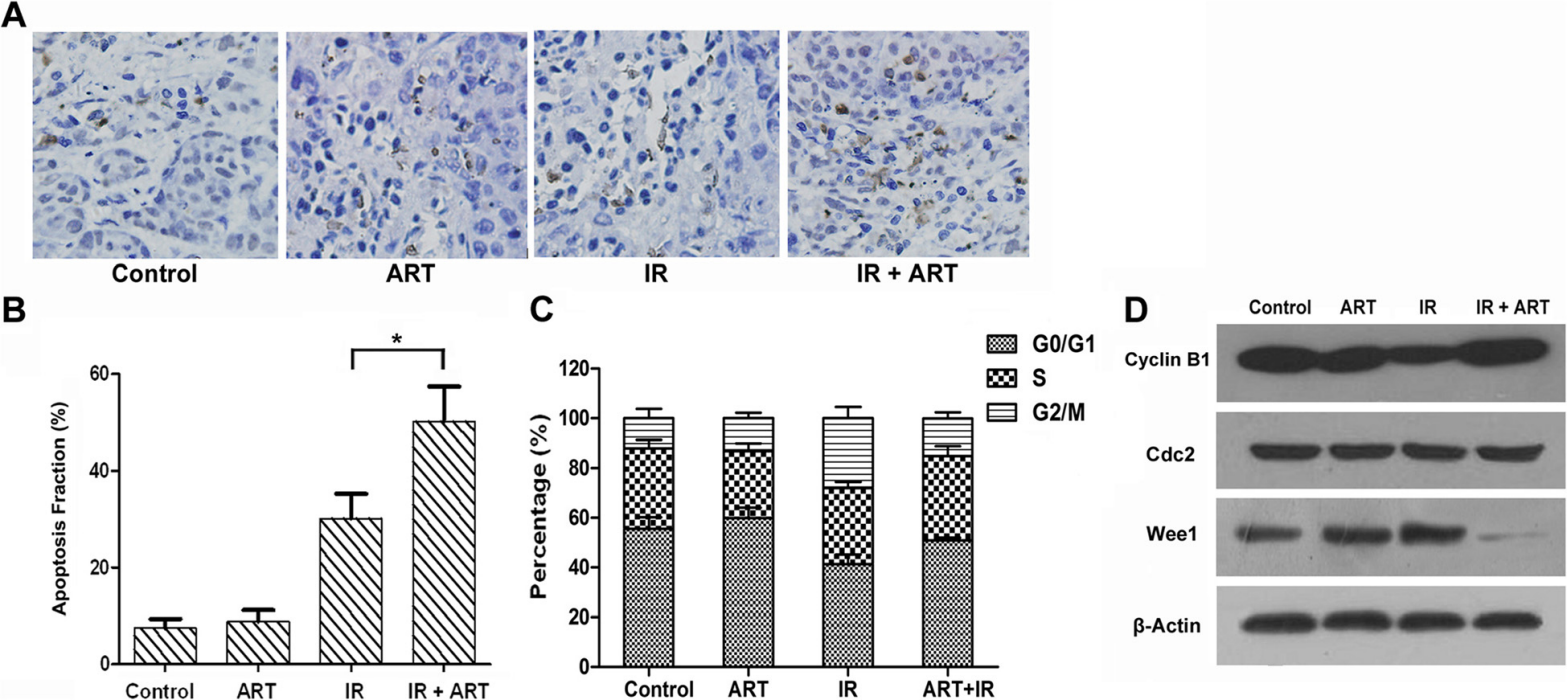
**The effect of ART on the apoptosis and cell cycle progression of HeLa xenografts. (A)** Representative TUNEL assays showing apoptosis in HeLa xenografts(magnification, × 400). **(B)** Percentage of TUNEL-positive cells per field are expressed as means ± SEM. *P < 0.05, compared with radiation alone group. **(C)** The cell cycle distributions of the xenografts were analyzed. Values shown are the mean ± SEM. **(D)** Western blot analysis of Cyclin B1, Cdc2 and Wee1 in the HeLa xenografts.

As shown in Figure 5A and 6B, mice treated with ART alone yielded similar results as the control group. Compared with the control group, the tumor volume of mice treated with radiation alone was reduced by 41.22%. Comparatively, HeLa xenografts that received combined treatment of 10 Gy radiation and ART exhibited much smaller tumors compared with mice that received radiation alone (volume reduction of 72.34% in IP + ART group), which suggests that ART can enhance radiosensitivity of HeLa xenografts. Consistent with *in vitro* studies, the radiosensitivity of SiHa xenografts was not significantly changed (volume reduction of 44.03% in IR alone group vs 48.79% in IR + ART group; *P* > 0.05)*.*

### ART reduces microvessel density, promotes apoptosis and inhibits G2-M phase arrest *in vivo*

To explore the mechanism for the tumor growth difference, the expression of CD31 which is an endothelial cell surface molecule that can be used to visualize microvessel density was detected by IHC. The results showed that xenografts from the control group or from ART treatment alone shared similar expression levels of CD31 (Figure 5C and D). Xenografts from the irradiation plus ART treatment group displayed significantly less positive staining for CD31 in HeLa xenografts (9.75% in IR + ART group *vs.* 24.25% in IR group, *P <* 0.05, Figure 5C) but not in the SiHa counterparts (Figure 5D). These results indicated that irradiation combined with ART influences the formation of microvessels and consequent tumor growth in HeLa xenografts.

We then measured cell apoptosis using the TUNEL assay to further investigate the apoptotic status in xenografts,. As shown in Figures 6A and B, irradiation plus ART resulted in significantly higher apoptotic cell death in the xenografts compared with IR alone group (50.30% in IR + ART group *vs.* 30.20% in IR group, *P <* 0.05, Figure 6B). These results indicated that irradiation combined with ART enhanced radiation-induced apoptosis in HeLa xenografts.

To determine whether irradiation plus ART influenced cell cycle progression in xenografts, flow cytometry was performed. The effect of combined radiation and ART treatment on the cell cycle in tumors is shown in Figure 6C. Treatment with ART alone leads to no observed changes while comparing with the control group. However, compared with the radiation group, the percentage of tumor cells in G1 phase was significantly increased by 9.52% (*P <* 0.05, Figure 6C) while the percentage of G2 phase was significantly decreased by 13.24% (*P <* 0.05, Figure 6C) in the radiation combined with ART group. It is likely that ART decreases G2 phase arrest of tumor cells after irradiation. We next examined the expression of Cyclin B1 and Cdc2 in the HeLa xenografts. As shown in Figure 6D, increased Cyclin B1 protein levels from xenografts were consistent with those in the previous *in vitro* study. However, the expression of Cdc2 was not changed in these xenografts, suggesting that the cell cycle changes may be triggered by Cyclin B1.

### ART increases radiosensitivity of HeLa cells via complex mechanisms

**Figure 7:**
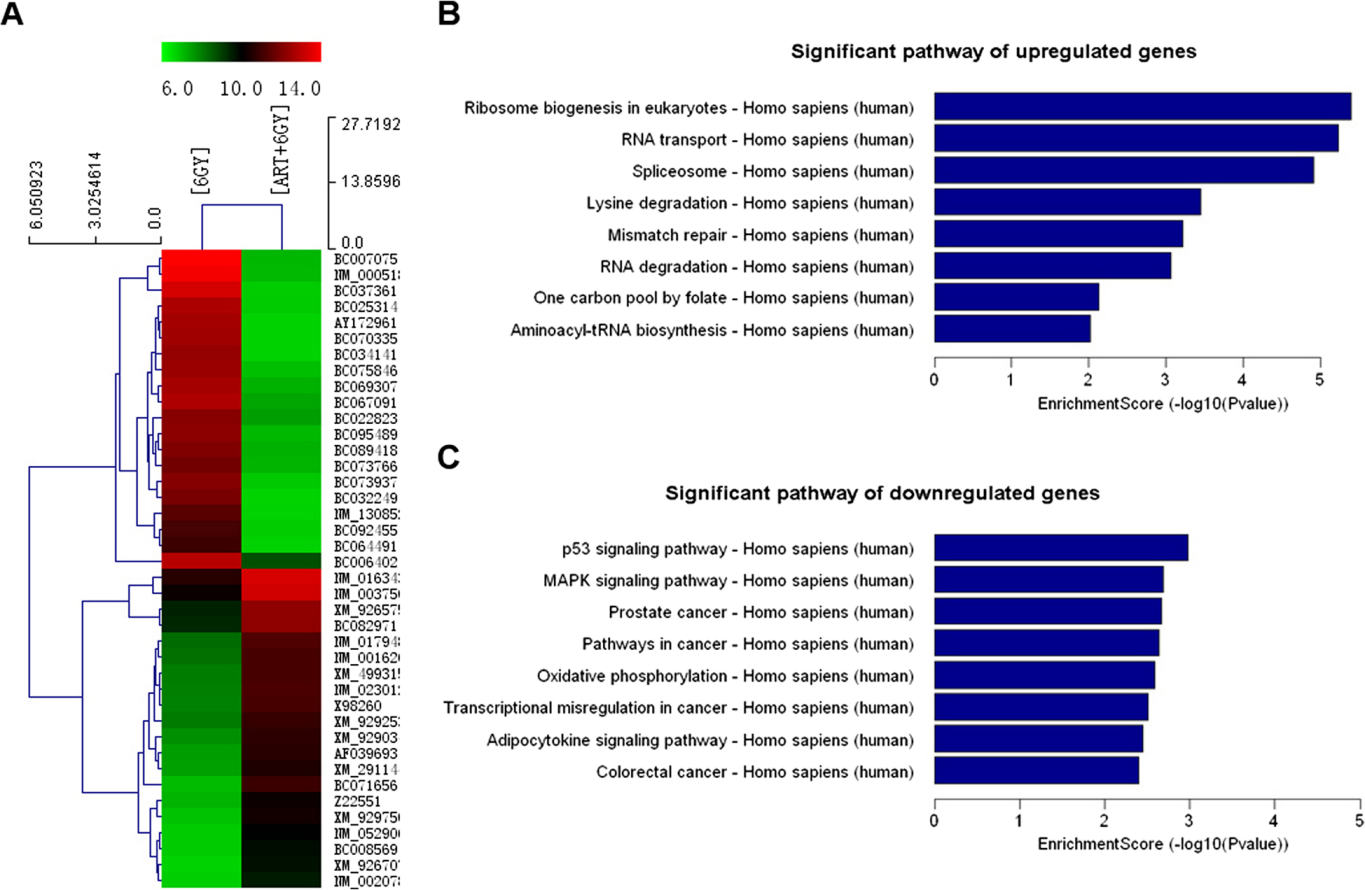
**Predicted significant pathways involved. (A)** Heat map of gene expression between HeLa cells after 6 Gy X-ray irradiation or 2.0 μmol/L ART prior to 6 Gy X-ray irradiation. **(B)** Predicted significant pathways for upregulated genes. **(C)** Predicted significant pathways for downregulated genes.

To further analyze the underlying mechanisms responsible for ART-mediated radiosensitivity, we profiled gene expression between HeLa cells after either 6 Gy X-ray irradiation or the combination of 2.0 μmol/L ART prior to 6 Gy X-ray irradiation. Twenty-four hours after the treatments, a total of 203 genes (91 upregulated and 112 downregulated genes) were identified with differential expression of 5-fold or more between the two groups of cells (Figure 7A and Table 1). Consistent with previous findings [19,20], the administration of ART remarkably inhibited the expression of genes associated with hemoglobin and immunoglobulin. As expected, ART seems to modulate the radiosensitivity of HeLa cells via complex mechanisms. Pathway analysis revealed that ART treatment affected multiple pathways including RNA transport, the spliceosome, RNA degradation, p53 signaling and MAPK (Figure 7B and C).

**Table 1:** **Microarray gene expression changes relative to irradiated HeLa cells (X-ray irradiation plus ART *vs. *** **X-ray irradiation alone)**

**Gene name**	**Fold change upregulated**	**Chromosome**	**Description**
IMAA	14.44	chr16	SLC7A5 pseudogene
HSUP1	10.05	chr20	Similar to RPE-spondin
FLJ11021	9.10	chr12	Similar to splicing factor, arginine/serine-rich 4
KIAA1904	8.98	chr22	KIAA1904 protein
ZRF1	8.95	chr7	Zuotin related factor 1
SDCCAG10	8.61	chr5	Serologically defined colon cancer antigen 10
LOC442573	8.47	chr7	Similar to postmeiotic segregation increased 2-like 2
EIF3S10	8.42	chr10	Eukaryotic translation initiation factor 3, subunit 10 theta
KTN1	8.34	chr14	Kinectin 1 (kinesin receptor)
LOC642617	8.23	chr2	Hypothetical protein LOC642617
LOC340089	7.98	chr5	Similar to nuclear pore membrane protein 121
GPR18	7.97	chr13	G protein-coupled receptor 18
LOC646074	7.93	chr22	Similar to nuclear pore membrane protein 121
NOL8	7.71	chr9	Nucleolar protein 8
AHNAK	7.26	chr11	AHNAK nucleoprotein (desmoyokin)
N/A	7.16	chr3	Homo sapiens cDNA clone MGC:99509 IMAGE:3939369
GOLGA4	7.10	chr3	Golgi autoantigen, golgin subfamily a, 4
LOC646316	6.95	chr4	Similar to Telomeric repeat binding factor 1 interacting protein 2)
CENPF	6.92	chr1	Centromere protein F, 350/400 ka (mitosin)
LOC643211	6.91	chr2	Hypothetical protein LOC643211
**Gene name**	**Fold change downregulated**	**Chromosome**	**Description**
HBB	116.84356	chr11	Hemoglobin, beta
HBB	110.22069	chr11	Hemoglobin, beta
IGHG1	93.40286	chr14	Immunoglobulin heavy constant gamma 1 (G1m marker)
N/A	60.31211	chr2	Anti-rabies SOJA immunoglobulin kappa light chain mRNA
IGHG1	59.625664	chr14	Immunoglobulin heavy constant gamma 1 (G1m marker)
IGKC	56.961563	chr2	Immunoglobulin kappa constant
IGKC	50.49672	chr2	Immunoglobulin kappa constant
HBD	43.09641	chr11	Hemoglobin, delta
IGHG1	42.666557	chr14	Immunoglobulin heavy constant gamma 1 (G1m marker)
IGHG1	40.846592	chr14	Immunoglobulin heavy constant gamma 1 (G1m marker)
IGKC	37.99056	chr2	Immunoglobulin kappa constant
IGHA1	36.901497	chr14	Immunoglobulin heavy constant alpha 1
IGKC	34.52912	chr2	Immunoglobulin kappa constant
IGLV3-25	28.34214	chr22	Immunoglobulin lambda variable 3-25
PLUNC	25.71026	chr20	Palate, lung and nasal epithelium carcinoma associated
IGLV3-25	25.19829	chr22	Immunoglobulin lambda variable 3-25
LOC652848	24.885628	chr14	Similar to Ig heavy chain V-II region ARH-77 precursor
N/A	19.36516	chr2	Homo sapiens cDNA clone MGC:104455 IMAGE:30352955
N/A	19.346416	chr22	Homo sapiens cDNA clone MGC:71261 IMAGE:4576612
IGHG1	17.280687	chr14	Immunoglobulin heavy constant gamma 1 (G1m marker)

## Discussion

The anti-malarial ART has been reported to act against a variety of cancer cells including leukemia, lung and colon cancer cell lines [21-23]. Previously, we have reported that artemisinin and its analog dihydroartemisinin are effective radiosensitisers for cervical cancer cells [9,10]. In this study, we found that another artemisinin derivative, ART, increased the radiosensitivity and promoted the apoptosis of p53-mutant HeLa cells but not of the wild-type p53 SiHa cells, both *in vitro* and *in vivo*. In HeLa cells, combined ART and radiation treatment decreased Wee1 but increased Cyclin B1 expression levels, impairing the irradiation-induced G_2_/M arrest. ART decreased the G2/M arrest induced by radiation, which most likely resulted in more irradiation-damaged cells entering mitosis. Moreover, the combined treatment of ART and irradiation increased apoptosis in cultured HeLa cells and HeLa xenografts. In the *in vivo* study, ART was given at a concentration of 100 mg/kg/day regularly for 7 days to achieve effective blood concentration in the nude mouse. Further investigation within other levels of ART should be tested.

ART has been reported to induce DNA damage response and repair genes, oncogenes, tumor suppressor genes and apoptosis-regulating genes [7]. DNA-damaging agents often induce cell cycle arrest in G1 or G2/M phases [24,25], which are facilitated by checkpoint mechanisms that provide time for the repair of sub-lethal DNA damage prior to the resumption of cell cycle progression [26]. Therefore, the abrogation of the G2 checkpoint, which promotes premature mitotic entry and subsequent cell death, has emerged as a potential therapeutic strategy [17,27]. Cells in G2/M phase are particularly susceptible to the effects of radiation. Because of this, agents that alter cell cycle progression are often potent radiation modifiers [28]. In eukaryotic cells, Wee1 phosphorylates Cdc2 on Tyr-15 and inhibits its kinase activity, thereby preventing entry into mitosis [29-32]. Therefore, suppression of Wee1 can reduce Cdc2 Tyr-15 phosphorylation and lead to the activation of Cdc2 kinase. Irradiation exposure can induce a G2/M arrest in HeLa cells through increased and decreased expression of Wee1 and Cyclin B1, respectively. We found that this cell cycle arrest and protein expression change was reversed by treatment with ART. The expression of Cdc2 exhibited no significant changes in either HeLa or SiHa cells that were treated with a combination of ART and irradiation, suggesting that ART might affect Cdc2 indirectly through Wee1. Wee1 is part of an intricate network of kinases and phosphatases that regulate the G2 checkpoint [33]; the abrogation of this checkpoint by Wee1 inhibition results in mitotic catastrophe. ART treatment resulted in similar abrogation of the radiation-induced G2 arrest in HeLa cells. However, ART elicited no significant inhibition effect on G1/S arrest, similarly to artemisinin [9,10].

In this study, we found that ART exhibits different radiosensitizing effects between HeLa and SiHa cells. ART is reported to act via p53-dependent and -independent pathways in cancer cells [22]. The tumor suppressor and transcription factor p53 is a major regulator of cellular defense against neoplastic transformation and cancer development [34-37]. Defects in p53-dependent pathways are correlated with tumor resistance to radiation and chemotherapy [35]. The present study used paired p53-positive and p53-negative cancer cells to confirm the hypothesis that abrogation of the G2 checkpoint by targeting Cyclin B1 and Wee1 kinases represents an effective therapeutic approach against p53-null cancer cells. Wild-type p53 might protect the genome from accumulating DNA damage and transmitting genetic mutations to subsequent daughter cells [34-36]. However, forced expression of wild-type p53 could partially change the radiosensitizing effect of ART in HeLa cells, which suggests that p53 status is only one of the reasons for the selective radiosensitization of ART. Such a phenomenon is also observed for artemisinin and dihydroartemisinin [9,10].

As expected, ART seems to modulate the radiosensitivity of HeLa cells via complex mechanisms by affecting multiple pathways including RNA transport, the spliceosome and RNA degradation. ART treatment also increased the transcripts of EIF3 (eukaryotic translation initiation factor 3), which induces apoptosis in multiple cancer cells [38]. However, we did not detect expression changes of Wee1 or Cyclin B1 in cells pretreated with ART, indicating that their expression changes may occur at the protein level.

In summary, ART enhances radiation-induced apoptosis and relieves the G2/M arrest *in vitro* and *in vivo*, which shows its promise as an effective radiosensitiser in cancer therapy. Clinical application of this anti-malarial drug can be expanded to complement radiotherapy for cancers.

## Abbreviations

ART: Artesunate; DHA: Dihydroartemisinin; SER: Sensitizer enhancement ratios; CENPF: Centromere protein F; EIF3: Eukaryotic translation initiation factor 3.

## Competing interests

The authors declare that they have no competing interests.

## Authors’ contributions

JL and WZ carried out the molecular biology studies and drafted the manuscript. YT performed part of the molecular biology and drafted the figures. HC, RJ and YZ performed the animal experiment. SZ and HY performed the statistical analysis. JC, XZ and ZL participated in study design and modified the manuscript. All authors read and approved the final manuscript.
